# Glucose Increases STAT3 Activation, Promoting Sustained XRCC1 Expression and Increasing DNA Repair

**DOI:** 10.3390/ijms23084314

**Published:** 2022-04-13

**Authors:** Griffin M. Wright, Natalie R. Gassman

**Affiliations:** 1 College of Medicine Depart of Physiology & Cell Biology, University of South Alabama, Mobile, AL 36688, USA; gmw1821@jagmail.southalabama.edu; 2 Mitchell Cancer Institute, University of South Alabama, Mobile, AL 36607, USA; 3 Department of Pharmacology and Toxicology, The University of Alabama at Birmingham, Birmingham, AL 35294, USA

**Keywords:** DNA repair, stress, XRCC1, glucose, DNA damage, STAT3

## Abstract

Dysregulation of DNA repair is a hallmark of cancer, though few cancer-specific mechanisms that drive the overexpression of DNA repair proteins are known. We previously identified STAT3 as a novel transcriptional regulator of X-ray cross-complementing group 1 (XRCC1), an essential scaffold protein in base excision repair in triple-negative breast cancers. We also identified an inducible response to IL-6 and epidermal growth factor stimulation in the non-tumorigenic embryonic kidney cell line HEK293T. As IL-6 and EGF signaling are growth and inflammatory-inducible responses, we examined if glucose challenge can increase STAT3 activation, promoting adaptive changes in XRCC1 expression in different cell types. Acute high glucose exposure promoted XRCC1 expression through STAT3 activation, increasing the repair of methyl methanesulfonate-induced DNA damage in HEK293T cells and the osteosarcoma cell line U2OS. Sustained exposure to high glucose promoted the overexpression of XRCC1, which can be reversed upon glucose restriction and down-regulation of STAT3 activation. Thus, we have identified a novel link between XRCC1 expression and STAT3 activation following exogenous exposures, which could play a critical role in dictating a cancer cell’s response to DNA-damaging agents.

## 1. Introduction

Changes in the balance of DNA repair proteins contribute to cancer initiation, progression, and treatment. Somatic and germline mutations, altered epigenetic regulation, and overexpression of DNA repair proteins are observed in various cancers, but the underlying mechanisms are just beginning to be unraveled. While dysfunction of DNA repair proteins through their loss or mutations has garnered significant research focus, factors driving the overexpression of DNA repair proteins and improving DNA repair capacity in cancer cells are not well understood. 

We have identified dysregulation of DNA repair proteins in triple-negative breast cancer (TNBC) cells that alter the DNA repair capacity of these cells [1,2]. We also recently linked overexpression of X-ray cross-complementing group 1 (XRCC1), a base excision repair (BER) protein, to the transcriptional activities of signal transducer and activator of transcription 3 (STAT3) [3]. BER is essential to repairing the myriad of DNA base lesions accumulated from endogenous and exogenous exposures daily. XRCC1 has no enzymatic function but serves as a scaffold protein facilitating the actions of other DNA repair proteins [4,5]. Although it lacks enzymatic activity, XRCC1 plays a critical role in efficient BER repair and deletion of *XRCC1* is embryonically lethal. In addition to its role in BER, XRCC1 has been shown to function in multiple other DNA repair pathways, including nucleotide excision repair through its interaction with the DNA ligase III α and double-strand break repair through its interactions with poly(ADP-ribose) polymerase 1 (PARP1) in alternative, non-homologous end-joining [6]. XRCC1 is dysregulated in multiple cancers beyond breast cancer [2]. XRCC1 overexpression has been shown to increase resistance to DNA-damaging chemotherapeutics in gastric and ovarian cancers, while low XRCC1 is correlated with hypersensitivity to DNA-damaging agents [7,8,9,10,11,12]. However, little is known about the transcriptional regulation of XRCC1 and the exposures that drive its dysregulation [13,14].

Since the discovery of the Warburg effect over a century ago, the role of glucose in cancer formation and progression has seen increased attention. Chronic inflammation and hyperglycemia result in an increased prevalence and mortality associated with many cancers, including breast and colorectal [15,16,17,18]. Inflammation and hyperglycemia also induce oxidative stress and DNA damage, which are significant factors contributing to disease development and progression [19,20]. While there has been considerable focus on changes in antioxidant systems and regulatory responses driven by NRF2 and NFκB, several studies have also noted DNA damage and repair changes [19].

Hyperglycemia causes DNA lesions and strand breaks and alters the DNA damage response in renal and prostate cancers [21,22]. Notably, a reduction in the expression of DNA repair proteins involved in nucleotide excision repair, homologous recombination, and mismatch repair were observed [21,22,23]. Chemo- and radiation-resistance were also noted in normal renal epithelial cells and renal cell carcinoma after high glucose exposure and attributed to altered DNA damage response and reduced repair, though DNA repair protein expression changes were not examined [21]. Alterations in the XRCC1 gene and protein expression were reported following glucose concentration changes in breast cancer cell lines and hepatocytes, suggesting differences in the response between tissue and cell types [10,24].

Our previous work demonstrated that STAT3 was a novel regulator of XRCC1 in TNBC cell lines, which have a constitutive activation of STAT3 [3,25]. STAT3 transcriptional activities are activated by phosphorylation at tyrosine 705 (Y705), leading to dimerization and translocation into the nucleus [26]. STAT3 activation following high glucose exposure has been documented in numerous cell lines [27,28]. We also found that STAT3 serves as an inducible regulator of XRCC1 in the non-tumorigenic human embryonic kidney cell lines HEK293T [3]. However, no links between increased glucose concentrations, the activation of STAT3, and increased XRCC1 and DNA repair have been made.

This study demonstrates that high glucose exposures drive XRCC1 expression through increased STAT3 activation. We show increased STAT3 activation and binding at the *XRCC1* promoter across cell line models following acute high glucose exposure, resulting in increased resistance to DNA damaging agents. Continuous exposure to high glucose concentrations promoted sustained STAT3 activation and XRCC1 expression, demonstrating that dysregulation of XRCC1 and DNA repair is achieved through STAT3 activation across different cell types.

## 2. Results

### 2.1. Acute High Glucose Stimulates Activation of STAT3 and XRCC1 Expression

**Figure 1:**
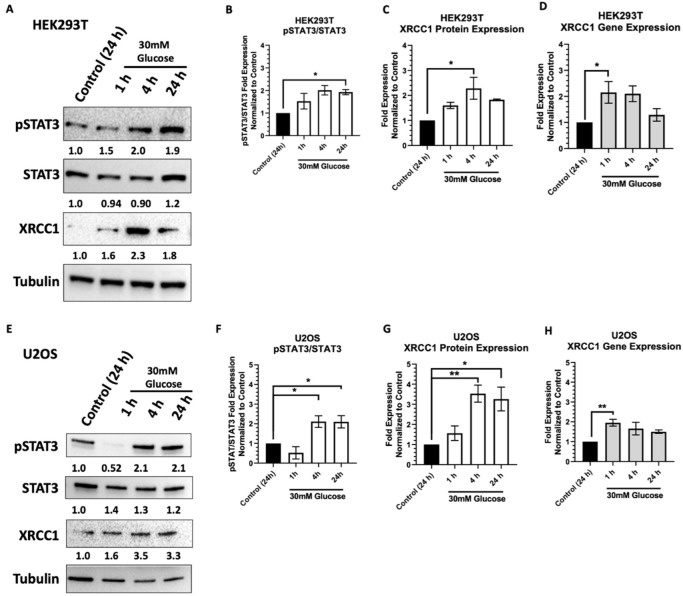
High glucose activates STAT3, subsequently increasing XRCC1 protein and gene expression. (**A**) Representative immunoblot showing increased XRCC1 following acute high glucose (30mM) for 1, 4, and 24 h in HEK293T, including mean values for three biological replicates. (**B**) pSTAT3 relative to total STAT3 in HEK293T normalized to loading control then control, untreated. (**C**) XRCC1 protein expression in HEK293T normalized to control. (**D**) *XRCC1* gene expression in HEK293T normalized to control. (**E**) Representative immunoblot showing increased XRCC1 following acute high glucose (30 mM) for 1, 4, and 24 h in U2OS, including mean values for three biological replicates. (**F**) pSTAT3 relative to total STAT3 in U2OS normalized as described in B. (**G**) XRCC1 protein expression in U2OS normalized to control. (**H**) *XRCC1* gene expression in U2OS normalized to control. * *p* < 0.05, ** *p* < 0.01.

To examine the high glucose regulation of XRCC1, we exposed cells to 30 mM glucose for up to 24 h. We selected the osteosarcoma cell line U2OS, which has high STAT3 activation, and HEK293T, which we showed previously has an inducible STAT3 response [3]. HEK293T cells were grown in 25 mM glucose (basal glucose (BG)), and the U2OS cells were grown in 11 mM (BG). Cells were then exposed to 30 mM high glucose (HG) through glucose addition to the basal medium for 1, 4, and 24 h. We observed an increase in STAT3 activation as indicated by an increase in STAT3 phosphorylation at Y705 in the HEK293T and U2OS cells (Figure 1A,B,E,F). In the HEK293T, phosphorylation of STAT3 started increasing 1 h after exposure and continued out to 24 h (Figure 1B). Similarly, XRCC1 gene expression increased at 1 and 4 h (2.2 ± 0.41 and 2.1 ± 0.30, respectively) following high glucose exposure, and the XRCC1 protein showed a sustained increase at 4 h (2.3 ± 0.44) (Figure 1C,D). In contrast, the U2OS cells showed an initial decrease in phosphorylation of STAT3 at 1 h of glucose exposure, but then pSTAT3 increased significantly at 4 h and was sustained at this higher level at 24 h (Figure 1F). The XRCC1 protein expression peaked at 4h and continued for 24 h (3.5 ± 0.42 and 3.3 ± 0.59, respectively), and XRCC1 gene expression increased only at 1h after high glucose exposure (2.0 ± 0.20, Figure 1G,H). To confirm that the increased XRCC1 expression was induced by glucose-induced pSTAT3, we co-exposed HEK293T cells to 30 mM glucose and 15 µM alantolactone, which blocks phosphorylation of STAT3 without significantly reducing the protein levels of STAT3, and the increase in XRCC1 expression was abrogated (Supplementary Figure S1).

### 2.2. STAT3 Occupancy of the XRCC1 Promoter Increases following Acute High Glucose Exposure

**Figure 2:**
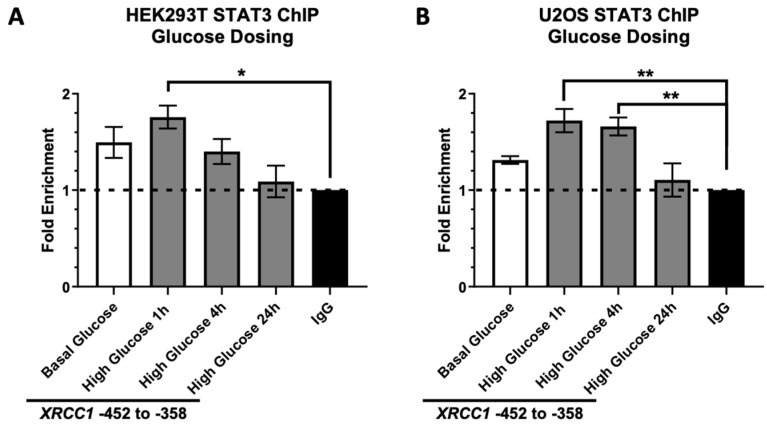
Increased STAT3 occupancy at the *XRCC1* promoter following acute high glucose exposure. ChIP analysis of STAT3 occupancy at the *XRCC1* promoter following acute high glucose (30 mM). exposure for 1, 4, and 24 h in HEK293T (**A**) and U2OS (**B**) cells normalized to respective IgG controls. * *p* < 0.05, ** *p* < 0.01.

To further confirm that acute high glucose increases XRCC1 expression through a STAT3 transcription mechanism, we performed chromatin immunoprecipitation (ChIP) to follow occupancy changes of STAT3 at the XRCC1 promoter with a 96 bp region (−452 to −358) previously identified as a STAT3 binding site within the XRCC1 promoter [3]. The occupancy of STAT3 at the XRCC1 promoter was assessed in HEK293T and U2OS cells following the 30 mM glucose exposure for 1, 4, and 24 h (Figure 2A). In the HEK293T, STAT3 occupancy of the XRCC1 promoter increased significantly 1 h following high glucose (1.8 ± 0.12, enrichment compared to IgG control) before lowering at 4 and 24 h. The U2OS cells showed a more sustained peak of STAT3 occupancy at the XRCC1 promoter 1 and 4 h following high glucose exposure (1.7 ± 0.12 and 1.7 ± 0.093, enrichment compared to IgG control), returning to a level slightly below basal glucose at 24 h (Figure 2B). These data indicated that STAT3 activation following acute high glucose exposure resulted in increased XRCC1 gene and protein expression through increased STAT3 occupancy at the XRCC1 promoter.

### 2.3. High Glucose-Induced XRCC1 Promotes Cell Survival through Increased DNA Repair 

**Figure 3:**
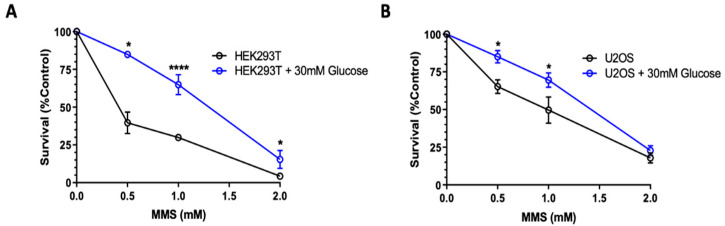
Increased MMS survival following acute high glucose exposure: survival curve of HEK293T (**A**) and U2OS (**B**) cells in basal glucose medium and with 30 mM glucose pre-treatment for 4 h, then co-exposure with MMS for 1 h, and continued exposure after MMS removal. The mean survival ± standard error of the mean (SEM) (%) normalized to 0 mM control is shown. * *p* < 0.05, **** *p* < 0.0001.

Increased XRCC1 expression is associated with increased survival against genotoxic stress [7]. We next examined the extent of high-glucose-induced XRCC1 expression altered cell survival after exposure to the BER alkylating agent methyl methanesulfonate (MMS). Cytotoxicity of MMS was assessed by cell growth inhibition. Cells were pre-treated with high glucose for 4 h before MMS exposure to ensure XRCC1 expression was increased (Figure 1). Cells were then exposed to increasing concentrations of MMS for 1 h in high glucose and sustained in high glucose after exposure (Figure 3). To confirm the changes in survival were not due to increased proliferation, the doubling time for acute high glucose exposure was assessed, and no significant difference in growth was seen (Supplementary Figure S2).

**Figure 4:**
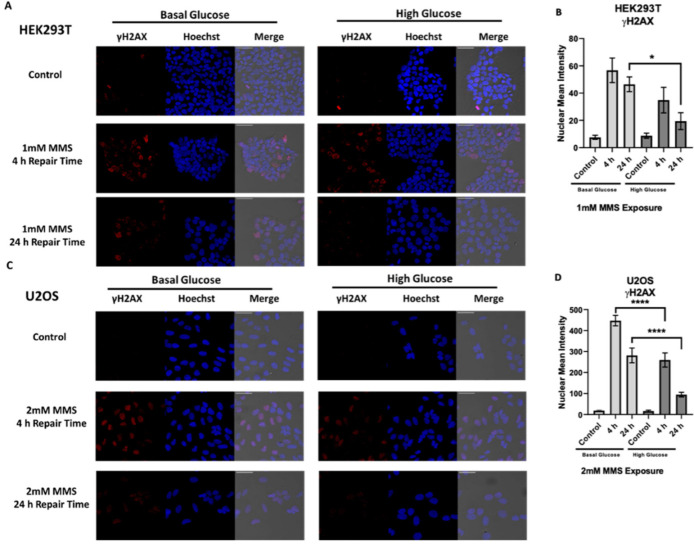
Acute glucose exposure reduced γ-H2AX signaling after MMS exposure, indicating decreased DNA damage signaling and resolution of DNA strand breaks. (**A**) Representative immunofluorescence images of basal glucose (BG) HEK293T and acute high glucose (HG) HEK93T 4 and 24 h post 1 mM MMS. (**B**) Nuclear mean fluorescent intensity of γ-H2AX in BG and HG HEK293T. (**C**) Representative immunofluorescence images of BG and acute HG U2OS 4 and 24 h post 2 mM MMS. (**D**) Nuclear mean fluorescent intensity of γ-H2AX in BG and HG U2OS. * *p* < 0.05, **** *p* < 0.0001. Scale bar is 100 µm.

To confirm the increased survival is due to changes in DNA repair, we examined changes in DNA damage signaling after MMS challenge in basal and acute high glucose conditions. HEK293T and U2OS cells were dosed with IC_25_ doses of MMS 1 and 2mM, respectively (Figure 3). Phosphorylation of H2AX at the serine 139 residue (γ-H2AX) was used to assess the formation of single and double strand breaks following exposure to MMS. In the HEK293T cells, a significant reduction in the γ-H2AX nuclear signal was seen 24 h post-MMS exposure in the high glucose cells compared to their BG controls (Figure 4A,B). The U2OS cells saw a more significant reduction in the γ-H2AX nuclear signal at 4 and 24 h post-MMS exposure than the BG controls (Figure 4C,D).

**Figure 5:**
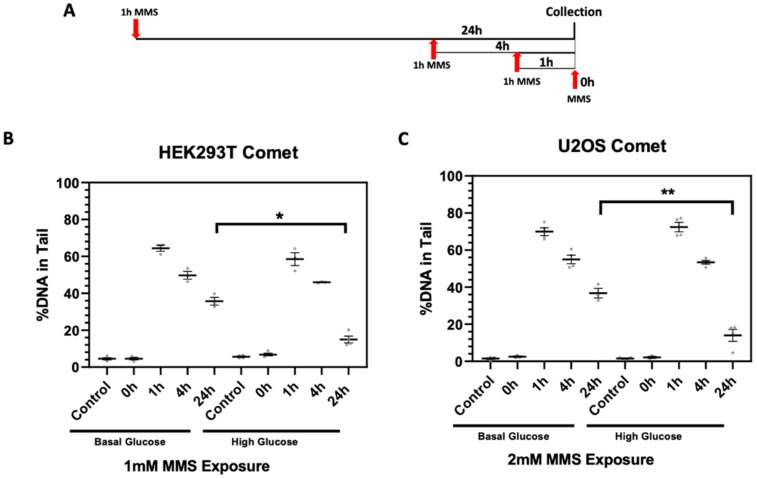
Comet assay showed increased DNA repair following acute high glucose exposure. (**A**) Timeline of MMS dosing for the comet assay. (**B**) The % DNA in the comet tail in basal glucose (BG) and acute high glucose (HG) HEK293T cells at 0, 1, 4, and 24 h repair times post-1 mM MMS exposure. (**C**) The % DNA in the comet tail in BG and acute HG U2OS cells 0, 1, 4, and 24 h post-2 mM MMS exposure. * *p* < 0.05, ** *p* < 0.01.

Finally, we examined DNA repair dynamics using alkaline single cell gel electrophoresis (comet assay) under basal and acute high glucose conditions. DNA repair was assessed by monitoring the percentage of DNA in the comet tail after 1 h exposure to 1 or 2 mM MMS (IC_25_) and 0, 1, 4, and 24 h of repair time in the HEK293T and U2OS cells, respectively (Figure 5A). Increased DNA (%) in the comet tail indicates the presence of DNA strand breaks and alkaline labile sites. Following MMS exposure, the acute high glucose, pre-treated HEK293T and U2OS cells showed an increase in DNA repair as indicated by the reduction of the percentage of DNA in the comet tail at the 24 h repair time following MMS exposure (17% ± 2.2% and 17% ± 5.7%, respectively) compared to their BG HEK293T and U2OS MMS-treated counterparts at the same 24 h time point (36% ± 6.3% and 37% ± 2.5, respectively) (Figure 5B,C, Supplementary Figure S3). 

### 2.4. Adaptive Changes in Glucose Concentration Alter STAT3 Activation, XRCC1 Expression, and STAT3 Occupancy of the XRCC1 Promoter

Next, we wanted to determine if adaptive changes due to long-term glucose concentration changes would drive dysregulation of XRCC1 in HEK293T and U2OS cells, similar to what we have previously reported in TNBC cells [3]. HEK293T and U2OS were adapted to low, physiologically relevant glucose (LG, 5 mM), basal glucose medium (BG, 25 mM for HEK293T and 11 mM for U2OS), and high glucose (HG, 30 mM) medium by passaging the cells for 1.5 weeks in increasing mixtures of glucose followed by growth in the final desired glucose concentrations for at least 2 weeks before analysis [29]. 

**Figure 6:**
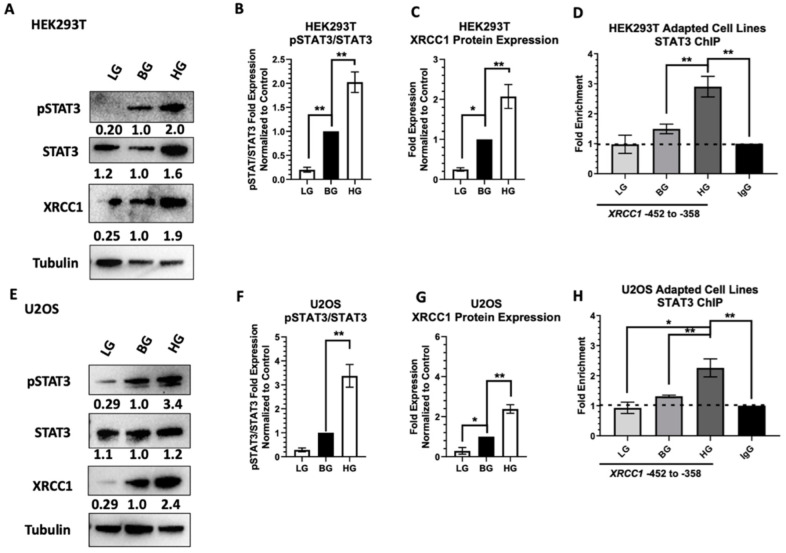
Alterations in XRCC1 expression following adaptation to different glucose concentrations. (**A**) Representative immunoblot of pSTAT3 and XRCC1 in HEK293T-adapted cell lines exposed to low glucose (5 mM, LG), basal glucose (25 mM, BG), and high glucose (30 mM, HG). (**B**) pSTAT3 relative to total STAT3 in HEK293T normalized to loading control then BG. (**C**) XRCC1 protein expression in HEK293T glucose-adapted cell lines normalized to BG. (**D**) ChIP analysis of STAT3 occupancy of at the XRCC1 promoter in glucose-adapted HEK293T cell lines normalized to respective IgG controls. (**E**) Representative immunoblot of pSTAT3 and XRCC1 in U2OS-adapted cell lines LG (5 mM), BG (11 mM), and HG (30 mM). (**F**) pSTAT3 relative to total STAT3 in U2OS normalized as in B. (**G**) XRCC1 protein expression in U2OS glucose-adapted cell lines normalized to BG. (**H**) ChIP analysis of STAT3 occupancy of the XRCC1 promoter in glucose-adapted U2OS cell lines normalized to respective IgG controls. * *p* < 0.05, ** *p* < 0.01.

Reduced STAT3 activation (lower pSTAT3) was seen for LG HEK293T (0.28 ± 0.79) and U2OS (0.34 ± 0.098) cells compared to their respective BG controls (Figure 6B,F). Sustained STAT3 activation occurred in HG HEK293T (2.4 ± 0.16) and U2OS (3.9 ± 0.10) cells compared to BG controls (Figure 6B,F). Sustained alteration in STAT3 activation resulted in dysregulation of XRCC1 protein expression changes with reduced XRCC1 (0.25 ± 0.04) in LG HEK293T and increased XRCC1 (1.9 ± 0.34) in HG HEK293T compared to the BG HEK293T controls (Figure 6C,G). Similar alterations occurred in U2OS cells with lower XRCC1 protein expression (0.29 ± 0.17) in LG U2OS and increased XRCC1 expression (2.4 ± 0.21) in HG U2OS than BG U2OS controls (Figure 6C,G). We also confirmed STAT3 occupancy at the XRCC1 promoter using ChIP. Decreases in STAT3 activation in LG HEK293T and U2OS cells correlated with a reduction below the BG control of STAT3 enrichment at the XRCC1 promoter (0.98 ± 0.30 and 0.93 ± 0.19, compared to IgG controls, respectively) (Figure 6D,H). Sustained activation of STAT3 in HG HEK293T and HG U2OS cells correlated with an increase of enrichment of STAT3 at the XRCC1 promoter (2.9 ± 0.34 and 2.3 ± 0.30, compared to IgG control, respectively) compared to BG HEK293T and U2OS cells (1.5 ± 0.16 and 1.3 ± 0.40, respectively) (Figure 6D,H). 

### 2.5. Low Glucose Adaptation of the TNBC Cell Line MDA-MB-231 Only Partially Reduces STAT3 Occupancy of the XRCC1 Promoter 

**Figure 7:**
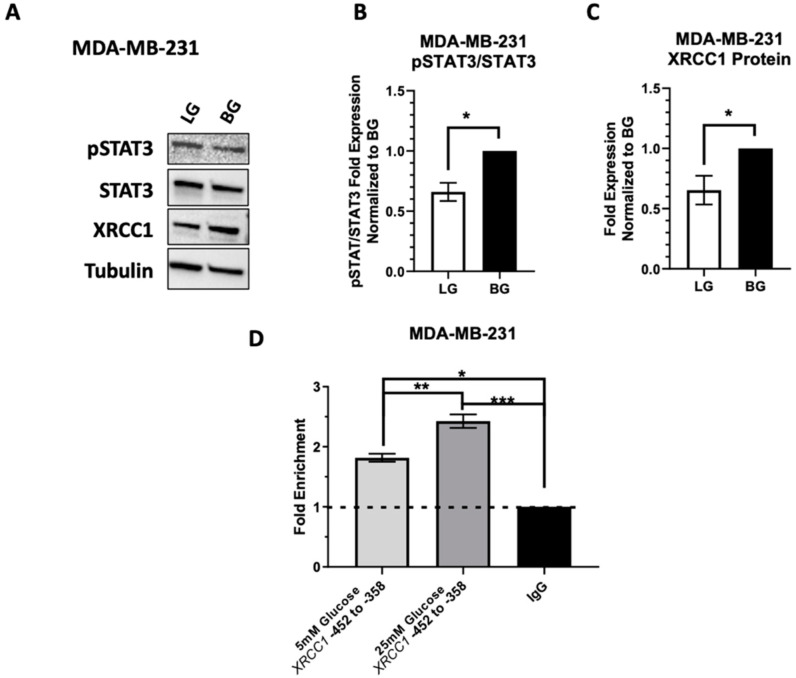
MDA-MB-231 pSTAT3 levels are resistant to decreased glucose concentrations. (**A**) Representative immunoblot of pSTAT3 and XRCC1 in MDA-MB-231-adapted cell lines exposed to low glucose (5 mM, LG) and basal glucose (25 mM, BG). (**B**) XRCC1 protein expression in glucose-adapted MDA-MB-231 cell lines. (**C**) pSTAT3 relative to total STAT3 in MDA-MB-231 normalized to loading control then BG. (**D**) ChIP analysis of STAT3 occupancy at the XRCC1 promoter in glucose-adapted MDA-MB-231 cell lines normalized to respective IgG controls. * *p* < 0.05, ** *p* < 0.01, *** *p* < 0.001.

We next wanted to see if glucose restriction altered the activation of STAT3 and regulation of XRCC1 in the MDA-MB-231 cells. We previously reported that STAT3 plays a major role in regulating XRCC1 in TNBC cell lines, including the MDA-MB-231 [3]. Given the constitutive activation of STAT3 in basal glucose (25 mM) MDA-MB-231, MDA-MB-231 cells were only adapted to low glucose-containing media (5 mM) as described above. Following the adaptation to LG, MDA-MB-231 showed only a slight reduction in the activation of STAT3 (0.71 ± 0.078), which resulted in a slight decrease of XRCC1 protein expression (0.65 ± 0.12) compared to the BG MDA-MB-231 control (Figure 7). Similarly, LG only slightly reduced the occupancy of STAT3 at the XRCC1 promoter (1.8 ± 0.066) compared to the BG control MDA-MB-231 (2.4 ± 0.11), and both the LG and BG MDA-MB-231 have a higher STAT3 occupancy at the promoter than that seen for BG HEK293T and U2OS cells (1.5 ± 0.16 and 1.3 ± 0.40, respectively).

### 2.6. IL-6 Expression and Stimulation Increases pSTAT3 and XRCC1 in HEK293T and U2OS

The observed sustained STAT3 activation under glucose restriction in MDA-MB-231 (Figure 7) compared to the HEK293T and U2OS cells (Figure 6) suggests that other factors influence the activation of STAT3 under glucose exposures. In humans, hyperglycemia increases inflammatory cytokines, such. as IL-6, a known activator of STAT3 [30]. Increasing glucose concentrations in cell culture medium has also been shown to increase IL-6 production and secretion across various cell types, including hepatocellular carcinoma and cholangiocarcinoma [31,32]. We previously demonstrated that the exogenous addition of IL-6 activated STAT3 and increased the expression of XRCC1 and the occupancy of STAT3 within the *XRCC1* promoter in HEK293T and MDA-MB-231 cells [3].

**Figure 8:**
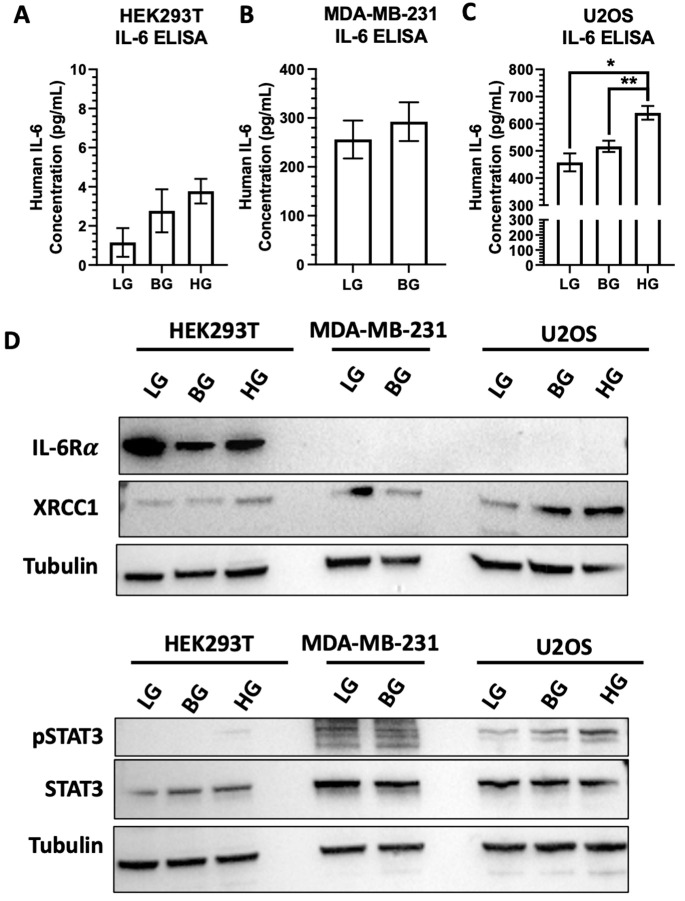
IL-6 secretion and IL-6Rα expression modulated STAT3 activation and XRCC1 expression. IL-6 concentration in the spent media of glucose-adapted HEK293T (**A**), MDA-MB-231 (**B**), and U2OS (**C**) cell lines. Representative immunoblot (**D**) of pSTAT3, STAT3, IL-6Rα, and XRCC1 in HEK293T-, MDA-MB-231-, and U2OS-adapted cell lines. * *p* < 0.05, ** *p* < 0.01.

With the variations in responses between the HEK293T, MDA-MB-231, and U2OS cells, we examined the endogenous IL-6 and IL-6R α levels and the effects of glucose stimulation (HG, 30 mM) and restriction (LG, 5 mM). We first measured IL-6 concentrations in the HEK293T, MDA-MB-231, and U2OS glucose-adapted cell lines (Figure 8A–C). HEK293T showed low IL-6 levels in the BG spent medium, while U2OS and MDA-MB-231 showed high IL-6 concentrations in the BG spent medium. Changing the glucose concentrations induced IL-6 release into the spent medium for U2OS cells, but only slight increases were observed in the HEK293T and MDA-MB-231 cells. Reducing the glucose in HEK293T and U2OS cells also reduced the IL-6 released into the medium (Figure 8A,C). We also examined the expression of IL-6R α under different glucose conditions across the cell lines (Figure 8D). IL-6 binding to IL-6R α activates STAT3 through several signaling mechanisms, including interactions with EGFR and JAK [26,33]. We observed high levels of IL-6R α in the HEK293T cells, and the receptor levels fluctuated with glucose concentrations. The high levels IL-6R α in the HEK293T cells made the immunoblot detection of the MDA-MB-231 and U2OS cells difficult. When the chemiluminescent exposure time was increased, the lowest expression of IL-6R α was seen in MDA-MB-231 cells with a higher expression of IL-6R α than was seen in the U2OS cells (Supplementary Figure S4).

**Figure 9:**
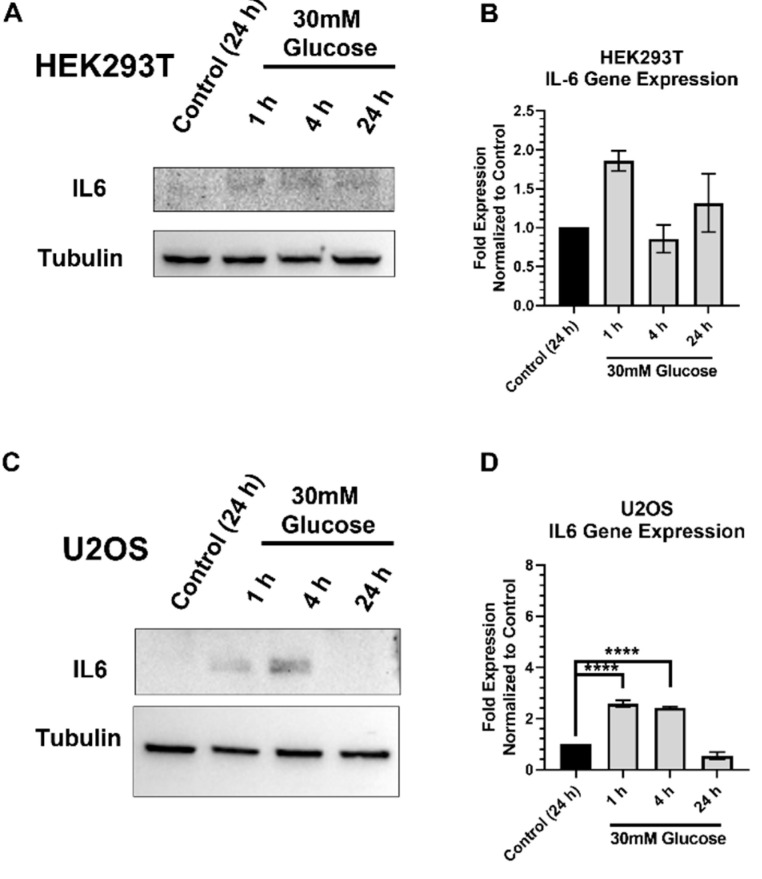
Acute high glucose stimulates IL-6 protein and gene expression: (**A**) Representative immunoblots showing increased IL-6 following acute high glucose 30mM for 1, 4, and 24h in HEK293T. (**B**) IL-6 gene expression in HEK293T normalized to control. (**C**) Representative immunoblot showing increased IL-6 following acute high glucose 30mM for 1, 4, and 24 h in U2OS. (**D**) IL-6 gene expression in U2OS normalized to control. **** *p* < 0.0001.

We then examined the timing and release of the IL-6 protein and its gene expression in HEK293T (Figure 9A,B) and U2OS (Figure 9C,D) cells following acute high glucose exposure to dissect its contribution to pSTA3 activation under high glucose exposures. We previously reported that IL-6 stimulated pSTAT3 and subsequently increased STAT3 occupancy at the XRCC1 promoter and increased XRCC1 expression in BG HEK293T and MDA-MB-231 cells [3]. We have also verified that IL-6 stimulated pSTAT3 and subsequently increased STAT3 occupancy at the XRCC1 promoter and increased XRCC1 expression in U2OS cells (Supplementary Figure S5 and Table S1). We saw peak expression of IL-6 within HEK293T cells after 1h of HG exposure and after 4h in U2OS cells (Figure 9A,C). We also saw alterations in the gene expression of IL-6 mRNA, and these changes were not significant in HEK293T cells, but were significant in the U2OS cells at 1 and 4 h after HG exposure (Figure 9B,D). Interestingly, the increases in IL-6 protein correlated well with the pSTAT3 elevation and XRCC1 protein increases in U2OS cells (Figure 1F,G), but they did not correlate with the increases in pSTAT3 and XRCC1 in HEK293T cells (Figure 1B,C).

Previously, when we directly exposed cells to 50 ng/mL IL-6, we only observed modest increases in STAT3 activation in MDA-MB-231 [3] and U2OS cells (Supplementary Figure S5 and Table S1). However, in HEK293T cells, increased inducibility of the pSTAT3-XRCC1 expression axis was observed following 50 ng/mL of IL-6 exposure [3]. These results suggest IL-6 plays some role in the glucose-driven activation of pSTAT3, but the extent of activation is dependent on IL-6 expression and secretion (Figure 8 and Figure 9). Therefore, IL-6 contributes to STAT3 activation in a cell-type-specific manner, but there are other driving factors for the activation of STAT3 and its subsequent increases in XRCC1.

### 2.7. High EGFR Expression in MDA-MB-231 Mitigates Glucose-Driven Changes in pSTAT3

**Figure 10:**
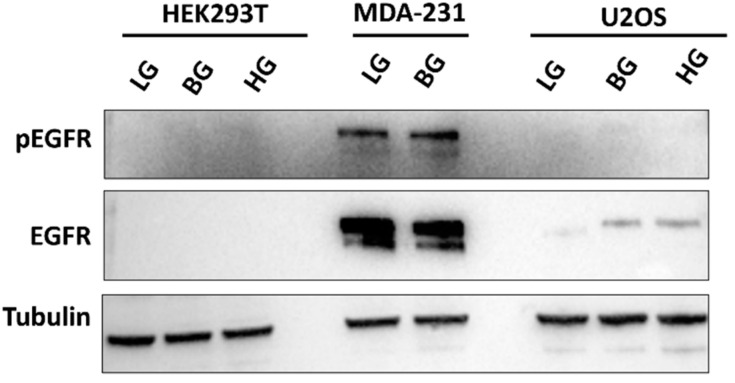
High expression of EGFR in MDA-MB-231 cells.Representative immunoblot of pEGFR (Y1068) and EGFR in HEK293T, MDA-MB-231, and U2OS glucose-adapted cell lines.

With the finding that other factors contribute to the activation of the pSTAT3 and increased expression of XRCC1, we re-examined the MDA-MB-231 cells to investigate why glucose restriction did not substantially reduce pSTAT3 and XRCC1 (Figure 7), similar to HEK293T and U2OS cells (Figure 6). There are several upstream regulators of STAT3, including epidermal growth factor (EGF), which we previously reported increased STAT3 activation and XRCC1 expression in MDA-MB-231 cells [3]. With the minimal IL-6 responses in MDA-MB-231 cells, we examined EGFR expression and activation across the cell line panel (Figure 10). Immunoblotting revealed increased EGFR expression in MDA-MB-231 cells, moderate EGFR expression in U2OS cells, and non-detectable EGFR expression in HEK293T cells (Figure 10). The high activation and expression of EGFR in the MDA-MB-231 cells contributes to the constitutive activation of STAT3 and elevated XRCC1 levels (Figure 7). More importantly, these levels remained high in the low glucose conditions, explaining the sustained activation of pSTAT3 and elevated XRCC1 under LG conditions compared to the HEK293T and U2OS cells (Figure 6 and Figure 7).

## 3. Discussion

We have previously reported that STAT3 drives the dysregulation of XRCC1 in TNBC while serving as an inducible transcription factor in the non-tumorigenic HEK293T cells [3]. Here, we showed that high glucose drives STAT3 activation, increasing the expression of XRCC1. Acute high glucose (30 mM) exposures increased STAT3 activation and XRCC1 protein and gene expression in the non-tumorigenic HEK293T and osteosarcoma U2OS cell lines. The XRCC1 gene and protein increases are linked to the increased occupancy of STAT3 at the *XRCC1* gene. Further, acute high-glucose-induced XRCC1 increased survival to DNA damage and improved repair dynamics in HEK293T and U2OS cells exposed to MMS.

Single-strand breaks can increase mutations and chromosomal aberrations if unrepaired, promoting carcinogenesis. Exogenous and endogenous agents can induce the expression of DNA repair proteins to respond to increased lesions and breaks to maintain genomic fidelity. The *XRCC1* regulator E2F1 behaves in this manner, increasing the expression of XRCC1 after exogenous insult with MMS to increase repair and maintain genomic fidelity [14]. Therefore, high DNA repair protein expression serves as a safeguard against carcinogenesis. However, DNA repair is a double-edged sword. The DNA itself is damaged during repair, and too much repair can also be lethal to the cell. Therefore, DNA repair proteins are tightly regulated to prevent extraneous repair. Again, Sp1 and ATM are examples of how this process works for XRCC1. If the repair load becomes too high, ATM phosphorylates Sp1, turning off *XRCC1* expression to prepare the cell for apoptosis [13]. In cancers, the processes by which DNA repair proteins become dysregulated are largely unknown. The overexpression of BER proteins has been correlated with a hypermutability phenotype that promotes cancer formation [34,35,36]. The overexpression of BER proteins is also correlated with chemotherapy and radiation therapy resistance, confirming that an imbalance of DNA repair proteins provides an advantage to cancer cells [7,8,9,12,37].

We first examined STAT3 regulation of XRCC1 in TNBC cell lines where STAT3 had become constitutively active, but the driving factors leading to dysregulation were not examined. Here, we confirmed that glucose challenge activated STAT3 and drove XRCC1 expression in cells without constitutively active STAT3, HEK293T, and U2OS. The temporal increase and decrease in STAT3 activation and XRCC1 expression after acute high glucose challenge in the HEK293T and U2OS cells demonstrate the inducible regulation of XRCC1 by STAT3 (Figure 1). However, when we adapted HEK293T and U2OS cells to high glucose continuously, we saw a sustained activation of STAT3 and continued expression of XRCC1, which suggests that continuous glucose exposure leads to increased XRCC1 protein levels. An advantage to increased XRCC1 expression is shown through improved cell survival and DNA repair after the MMS challenge (Figure 3 and Figure 5). DNA-damaging agents are commonly used in cancer therapy and the altered expression of XRCC1 has been found to modulate responses in numerous cancers [7,8,11,12,37,38,39]. STAT3 was not previously identified as a regulator of XRCC1, but the data here demonstrate that STAT3 activation promotes resilience to DNA damage, which likely contributes to chemoresistance or even radiotherapy resistance in cancer cells.

Critically, these effects could be reversed when activated STAT3 is lowered through glucose restriction. Low glucose media (5 mM) reduced STAT3 activation in HEK293T and U2OS cells, reducing the STAT3 enrichment at the XRCC1 promoter and XRCC1 gene and protein expression compared to the higher glucose media compositions. However, in MDA-MB-231 cells, low glucose adaptation resulted in a less significant reduction of STAT3 activation (Figure 7), STAT3 enrichment at the *XRCC1* promoter, and XRCC1 protein expression. When we examined the upstream regulators of STAT3 activation in these cell lines, the MDA-MB-231 cells, unlike the U2OS and HEK293T cells, also had highly activated EGFR, which likely maintains the STAT3 activation and elevated XRCC1 expression. Elevated EGFR is also found in another TNBC cell line, MDA-MB-468, which we previously demonstrated has constitutive STAT3 activation, high STAT3 occupancy at the *XRCC1* promoter, and elevated XRCC1 expression [3].

Beyond EGFR, we also noted clear differences in the expression of IL-6R α and the stimulation of IL-6 release across the cell line models. In particular, glucose stimulation significantly alters the expression of IL-6 in U2OS cells, and the increases in IL-6 correlate well with increased pSTAT3 and XRCC1 (Figure 1 and Figure 9). However, HEK293T cells do not have a significant IL-6 response when elevated pSTAT3 and XRCC1 are observed (Figure 1, Figure 8 and Figure 9). Interestingly, there is a high level of IL-6R α in HEK293T cells (Figure 8), and they show an inducible response to direct IL-6 stimulation [3], but this effect is not dominant in glucose exposures. Together, these findings suggest that multiple mechanisms drive the activation of STAT3 across different cell types. Still, cancer cells appear to be more responsive to elements that activate STAT3, and once STAT3 is activated, XRCC1 gene and protein levels are elevated and impact the cells **’** responses to DNA damage.

Chronic inflammation, increased inflammatory cytokine signaling, and increased mitogenic signaling are all common in cancer. Here, we showed that STAT3 activation induces XRCC1 expression, and continued activation of STAT3 drives BER dysregulation in non-tumorigenic HEK293T and tumorigenic U2OS cells. Additionally, the induction of this response following exogenous exposures alters the response to the DNA-damaging agent MMS and improves cell survival. These results are consistent with previous reports of chemoresistance when XRCC1 protein levels are elevated [7,8,11,12,37,38,39], but for the first time, we have linked the XRCC1 increases to activated STAT3.

Critically, we have determined that elevated pSTAT3 and XRCC1 can be mitigated with glucose restriction if other drivers of dysregulated STAT3 activation are absent. These findings suggest that the degree to which the effects of elevated XRCC1 can be reserved through therapeutic intervention upstream of STAT3 would depend highly on the signaling nodes used to activate STAT3. A better therapeutic strategy for reversing the dysregulation of BER observed here would be targeting STAT3, which we previously demonstrated with alanolactone and shRNA in TNBC cell lines [3].

In sum, this study identified a new transcriptional regulation mechanism for XRCC1, providing a novel link between exogenous exposures, activation STAT3, and DNA repair. This regulatory mechanism could have major implications in promoting genomic instability and modulating therapeutic response, both critical in the formation and progression of cancer. This work also suggests that the transcriptional regulation of BER proteins is highly responsive to exogenous and endogenous changes. Much more work is needed to understand known regulators, such as E2F1 and STAT3, and even unknown regulators, that respond to exogenous and endogenous signals and potentially interact with them [40]. BER factors, including PARP1, POL β, and XRCC1, are frequently dysregulated in cancers and associated with poor survival outcomes. We need a better understanding of the regulation landscape driving these changes to improve therapeutic targeting and patient survival.

## 4. Materials and Methods

### 4.1. Cell Culture 

HEK293T, U2OS, and MDA-MB-231 cells were purchased from the American Type Culture Collection (ATCC CRL-3216, HTB-26, and HTB-96, respectively; Manassas, VA, USA) within the previous 24 months and passaged < 10 times for all experiments (passage numbers from 6–15). HEK293T cells were grown in DMEM high glucose plus L-Glutamine supplemented with 1% sodium pyruvate (Hyclone, Logan, UT, USA, #SH30022.01) and 10% fetal bovine serum (FBS, Premium Select, R&D systems, Minneapolis, MN, USA). U2OS cells were grown in RPMI 1640 with L-glutamine (Corning, Glendale, AZ, USA, 10-040-CV) supplemented with 10% FBS. MDA-MB-231 cells were grown in DMEM high glucose plus Glutamax (Life Technologies, Carlsbad, CA, USA, #10566016) supplemented with 1% sodium pyruvate and 10% FBS. Biweekly testing for mycoplasma contamination was performed using the Lonza MycoAlert (Lonza, Bend, OR, USA, #LT07-318).

Low-glucose-adapted HEK293T and MDA-MB-231 cells were grown in DMEM low glucose and pyruvate (Thermo Fisher Scientific, Waltham, MA, USA, #11885084) supplemented with L-glutamine, 1% sodium pyruvate, and 10% FBS. Low-glucose-adapted U2OS cells were grown in RPMI 1640 with L-glutamine without glucose (Life Technologies #11879020), supplemented with 5mM glucose (D-(+)-Glucose Solution Sigma, St. Louis, MO, USA, #G8644) and 10% FBS.

### 4.2. Immunoblot

Immunoblot was performed as previously described. Briefly, cells were grown in 150 mm dishes and grown to 80% confluency. 1 × PBS was used to rinse cells before cells were scraped and pelleted at 1000 rpm and stored overnight at −80 °C. Pellets were then lysed, protein content was quantified by a Bradford assay, and 20 µg of lysate was separated on 4–15% SDS Page gel (Bio-Rad, Hercules, CA, USA, #4561084), and the cells were transferred to nitrocellulose membranes (Bio-Rad #1704156). The nitrocellulose membranes were blocked for 1 h at RT in 5% non-fat dry milk in Tris-buffered saline (VWR, Radnor, PA, USA, #J640-4L) containing 0.1% Tween20 (Thermo Fisher Scientific #BP337, TBS-T). The following primary antibodies were incubated overnight: XRCC1 (1:1000 #MS434P1, Fisher Scientific), pSTAT3 Y705 (1:500 Cell Signaling Technology, Danvers, MA, USA, #9131), STAT3 (1:1000 Cell Signaling Technology, #9139), pEGFR (1:500 Cell Signaling Technology, #3777), EGFR (1:1000 Cell Signaling Technology #4267), and IL-6R α (1:1000 Cell Signaling Technology #39837). Blots were incubated with horseradish peroxidase (HRP)-labeled secondary antibodies (goat anti-rabbit-HRP #7074 or goat anti-mouse-HRP #7076S from Cell Signaling Technology). A WesternBright Sirius (Advansta, San Jose, CA, USA, #K-12043) was used to detect target proteins. Immunoblotting was performed with three biological replicates. Protein levels were quantified relative to tubulin in each lane then normalized to the control, untreated lane using Image Lab Software (Bio-Rad). Phosphorylated STAT3 was normalized to total STAT3 and then normalized to the control, untreated lane.

### 4.3. Gene Expression Analysis 

Isolation of mRNA using an Invitrogen Cell to C_t_ kit (Life Technologies #4399002) was used for the relative gene expression in HEK293T and U2OS cells, following the manufacturers’ recommendations as previously described [3]. Cells were plated in 96-well plates and grown to 70% confluency before treatment with high glucose media. Cells were then lysed, and RT-PCR was performed to produce cDNA. Following cDNA synthesis, qPCR was performed using the appropriate TaqMan gene expression primers (XRCC1 Hs00959834_m1 FAM and IL-6 Hs00174131_m1 FAM) and TaqMan master mix. Each gene expression experiment was performed in technical triplicates with three biological replicates. Quantifications are represented as the mean of the three biological replicates ± the standard error of the mean (SEM).

### 4.4. Chromatin Immunoprecipitation

Chromatin immunoprecipitation was performed as previously described [3]. Briefly, cells were plated in 150 mm dishes and grown to 80% confluency. Cells were crosslinked in 1% formaldehyde in 1 × phosphate-buffered saline (PBS) at RT for 8–10 min. Cells were then lysed in 1 mL of Farnham lysis buffer (5 mM HEPES pH 8.0, 85 mM KCl, 0.5% NP-40) for 20 min on ice, followed by centrifugation at 2000 rpm to pellet the lysed sample. The pellet was then re-suspended in RIPA buffer (50 mM Tris-HCl pH8, 150 mM NaCl, 1% sodium deoxycholate, 1 mM EDTA, 0.1% SDS, 1% Triton X-100) for 20 min, followed by sonication on ice at an amplitude of 10 on a Misonix S-4000 with 15 s on/50 s off for a total time of 3.5 min for HEK293T and U2OS cells. Immunoprecipitation was performed by incubating chromatin with anti-STAT3 diluted to the manufacturers’ recommendation (Cell Signaling Technology #9131S) and Protein A/G magnetic beads (Thermo Fisher Scientific #88802) overnight at 4 °C with agitation on a rotator. Magnetic-bead-conjugated chromatin was then washed with an ice-cold LiCl wash buffer (100 mM Tris-HCl, 500 mM LiCl, 1% NP-40, 1% Triton X-100) and TE buffer (10 mM Tris-HCl pH 7.5, 0.1 mM EDTA). Proteinase K was added in ChIP elution buffer (1% SDS, 0.1M NaHCO_3_) and incubated at 65 °C with agitation (950 rpm) for 2 h, followed by proteinase K inactivation at 90 °C for 10 min. Isolated DNA was purified using a PureLink PCR purification kit (Life Technologies #K310002). XRCC1 primers were used as previously described [3].

### 4.5. Cell Growth Inhibition

Cells were plated at a density of 1 × 10^4^ (HEK293T and U2OS) and 2 × 10^4^ (MDA-MB-231) in 12-well dishes and allowed 48 h to adhere. Cells were pre-treated with basal glucose media with additional 5 mM glucose added for 4 h before challenging with MMS. Cells were exposed to MMS for 1 h at 0.5 mM, 1 mM, and 2 mM concentrations. MMS was washed using 1 × PBS, and fresh high glucose media was added. Cells were allowed to recover for 5 days before being trypsinized and resuspended in 1 mL of PBS. Resuspended cells were counted using a BioRad TC-10 Automated Cell Counter. Cell counts were performed in triplicate and normalized to control wells for a total of three biological replicates. Values were plotted as mean ± standard error of the mean (SEM).

### 4.6. Immunofluorescence 

Immunofluorescence was performed as previously described [2]. Briefly, cells were plated in 60 mm fluorodishes at 2.5 × 10^4^ per plate, allowing 24 h for cells to adhere. For HEK293T, dishes were pre-treated with poly-L-lysine solution (Sigma #P4707-50mL) for 5 min and allowed to dry for 1 h before cell plating. Cells were pre-treated with high-glucose-containing media for 4 h before MMS exposure. Cells were exposed to MMS-containing media for 1 h, and the cells were then washed with 1 × PBS and allowed to recover in fresh high-glucose-containing media for 4 and 24 h. Dishes were fixed using 3.7% formaldehyde in 1 × PBS. Nuclear permeabilization was accomplished by Permeabilization Buffer (Biotium, Fremont, CA, USA, #22016) for 10 min incubation at RT. Dishes were then blocked using 2% BSA in PBS for 30 min at RT. Anti-Phospho-Histone H2AX (Ser139) (EMD Millipore, Burlington, MA, USA #05-636) primary antibody diluted in 2% BSA in PBS (1:400) was then incubated for 1h at RT. Dishes were washed with 1 × PBS and Alexa Fluor™ 546 goat anti-mouse IgG (H+L) (Thermo Fisher Scientific #A11003) secondary antibody was applied (1:2000) for 1 h. Nuclei were stained with Hoechst 33,342 (Thermo Scientific #62249) for 15 min at RT. Dishes were mounted in Prolong™ Gold Antifade Reagent (Life Technologies #P36930) and stored for up to 2 weeks before imaging. Fluorescence images were acquired using a Nikon A1 scanning confocal microscope with a Plan-Apochromat 20×/0.75 objective. To quantify mean intensity in the nucleus, a region of interest (ROI) generator was used to detect nuclei automatically in the DAPI channel. The nuclear mean intensity of γ-H2AX was exported for analysis.

### 4.7. Single Cell Gel Electrophoresis (Comet Assay)

Cells were plated in 96-well plates at a density of 2.5 × 10^4^ per well, allowing 24 h for the cells to adhere. Cells were pre-treated with high glucose media 4 h before MMS exposure. IC_25_ concentrations were used to determine MMS dosing. Cells were exposed to MMS containing media for 1 h, then media was removed, the wells were washed, and fresh media was added. After the indicated repair time, media was removed, and cells were trypsinized and resuspended. 100 µL of resuspended cells were then transferred to a CometChip **^®^** (Trevigen, Rockville, MD, USA, #4260-096-01). Following overlay with 0.75% low melting temperature agarose, the embedded CometChip **^®^** cells were lysed overnight at 4 °C in CometAssay lysis solution (Trevigen #4250-010-01). CometChips were then incubated in alkaline solution (200 mM NaOH, 1 mM EDTA, 0.1% Triton 100×) for a total of 40 min at 4 °C. Electrophoresis was then performed for 50 min at 22V. The alkaline solution was neutralized using 400 mM Tris for a total of 30 min at 4 °C followed by 20 mM Tris for a total of 30 min at 4 °C. CometChips **^®^** were then stained in SYBR™ Gold Nucleic Acid Gel Stain (Thermo Fisher Scientific #S11492) in 20 mM Tris for 1 h at 4 °C, followed by a 30 min de-stain in 20 mM Tris at 4 °C. CometChips were imaged using a Celigo S Imaging Cytometer (Nexcelom Bioscience, Lawrence, MA, USA). Acquired images were analyzed using Trevigen CometChip **^®^** analysis software.

### 4.8. Cell Adaptation

Basal glucose HEK293T and MDA-MB-231 cells were grown in 25 mM glucose-containing DMEM, and U2OS cells were grown in 11 mM containing RPMI. Adaptation to low glucose (5 mM) medium was accomplished in HEK239T and MDA-MB-231 cells by briefly growing the cells in 17 mM glucose-containing DMEM for 3 days. The media was then changed to 10 mM glucose-containing media for 1 week. Finally, cells were grown in low glucose (5 mM) DMEM for 1.5 weeks before being frozen and stored in liquid nitrogen. Similarly, U2OS cells were adapted to low-glucose-containing RPMI by growing cells in 7 mM containing RPMI for 6 days before changing the media to low glucose RPMI (5 mM) for 1.5 weeks before being frozen and stored in liquid nitrogen. High glucose adaptation was accomplished similarly. First, HEK293T and MDA-MB-231 ce4lls were grown in 27.5 mM containing DMEM for 1 week before being grown in high glucose (30 mM) DMEM for 1.5 weeks. Following adaptation, cells were frozen and stored in liquid nitrogen. Finally, U2OS cells were adapted to high-glucose-containing RPMI (30 mM) by briefly growing cells in 20 mM RPMI for 3 days, followed by 1 week of growth in 25 mM RPMI. Finally, cells were grown in high glucose RPMI (30 mM) for 1.5 weeks before being frozen and stored in liquid nitrogen. HEK293T cells were adapted from passage 8. Low and high glucose HEK293T-adapted cells were frozen at passage 11. U2OS cells were adapted from passage 8. Low glucose and high glucose U2OS cells were frozen at passage 10. MDA-MB-231 cells were adapted from passage 6. Low glucose MDA-MB-231 cells were frozen at passage 11.

### 4.9. IL-6 ELISA Quantification

IL-6 media concentrations were measured using the Quantikine **^®^** ELISA Human IL-6 kit (R&D systems, Minneapolis, MN, USA, #S6050), following the manufacturer’s instructions. Briefly, cells were plated in 150 mm dishes and allowed to grow to 70–80% confluency before media was collected. The ELISA was performed in technical triplicates, with the mean ± standard error of the mean (SEM) of three biological replicates. 

## Data Availability

All data is contained within the manuscript.

## Supplementary Materials

The following supporting information can be downloaded at: https://www.mdpi.com/article/10.3390/ijms23084314/s1.
