# Supplementary material for: Glucose Increases STAT3 Activation, Promoting Sustained XRCC1 Expression and Increasing DNA Repair
Source: Int J Mol Sci. 2022 Apr 13;23(8):4314. doi: 10.3390/ijms23084314 (PMC9029887; doi:10.3390/ijms23084314)
Supplement: Supplementary file 1 [file ijms-23-04314-s001.zip › ijms-1646148-supplementary.pdf]

Supplementary Information:

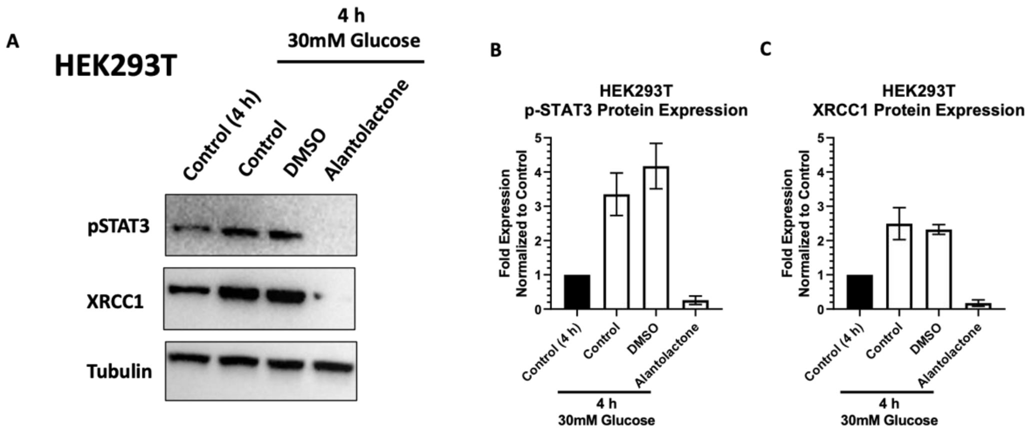

Figure S1. Alantolactone reverses acute high glucose induced increases in XRCC1 expression (**A**) Representative immunoblot for pSTAT3 and XRCC1 following 30 mM high glucose for 4h and 4h 30mM high glucose with 15 µM alantolactone co-exposure. Tubulin is the loading control. (**B**) pSTAT3 protein for control, control 4 h 30 mM glucose, DMSO 4 h 30 mM glucose, and 4 h 30 mM glucose with 15 µM alantolactone co-exposure (n = 2). pSTAT3 levels are normalized to the loading control, then to the control, untreated. (**C**) XRCC1 protein for control, control 4 h 30 mM glucose, DMSO 4 h 30 mM glucose, and 4 h 30 mM glucose with 15 µM alantolactone co-exposure (n = 2).

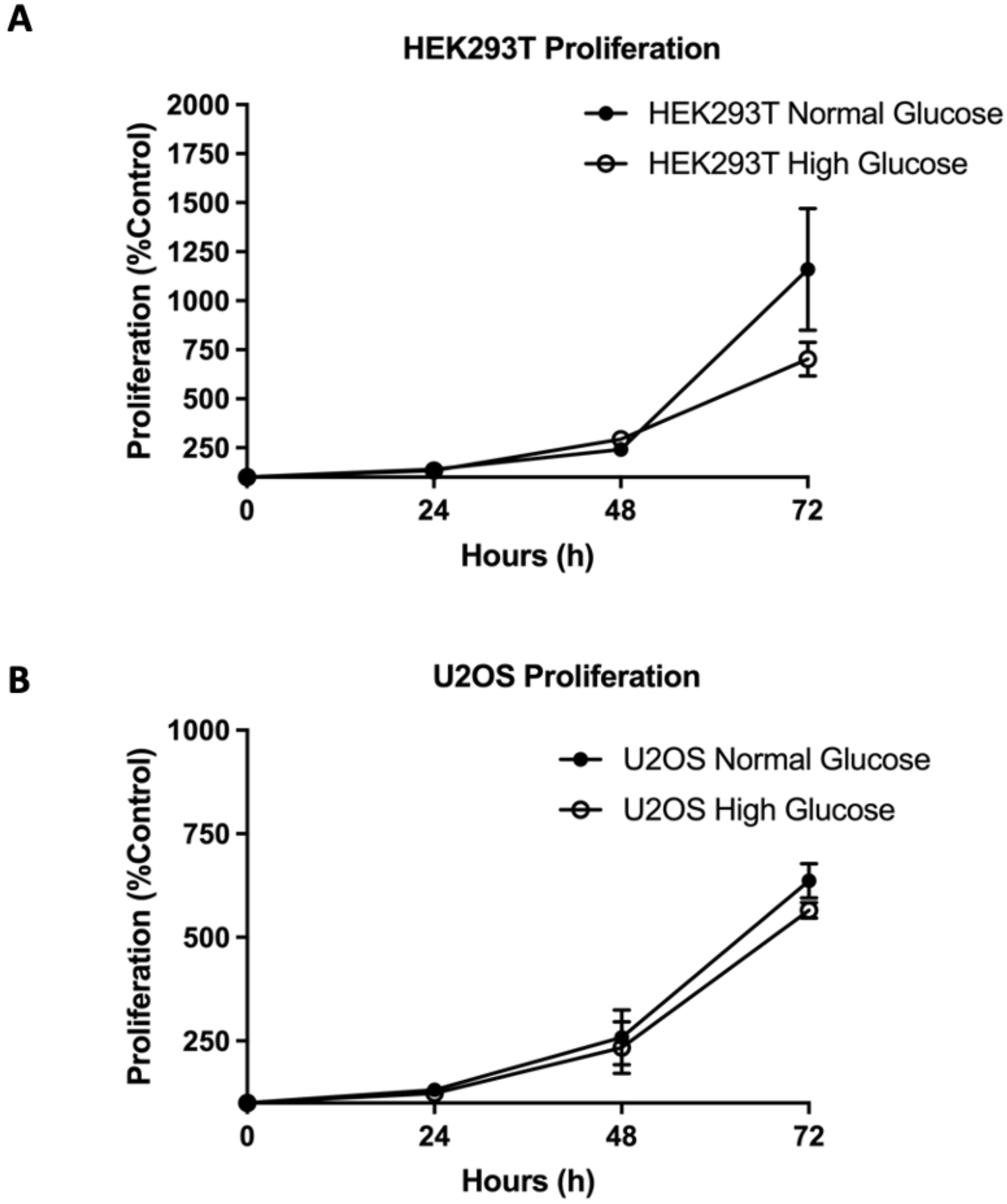

Figure S2. Doubling time for HEK293T (**A**) and U2OS (**B**) in basal glucose and high glucose is similar up to 72 h. Values presented as the mean cell count % of control ± the standard error of the mean (SEM) at each time point (*n* = 3).

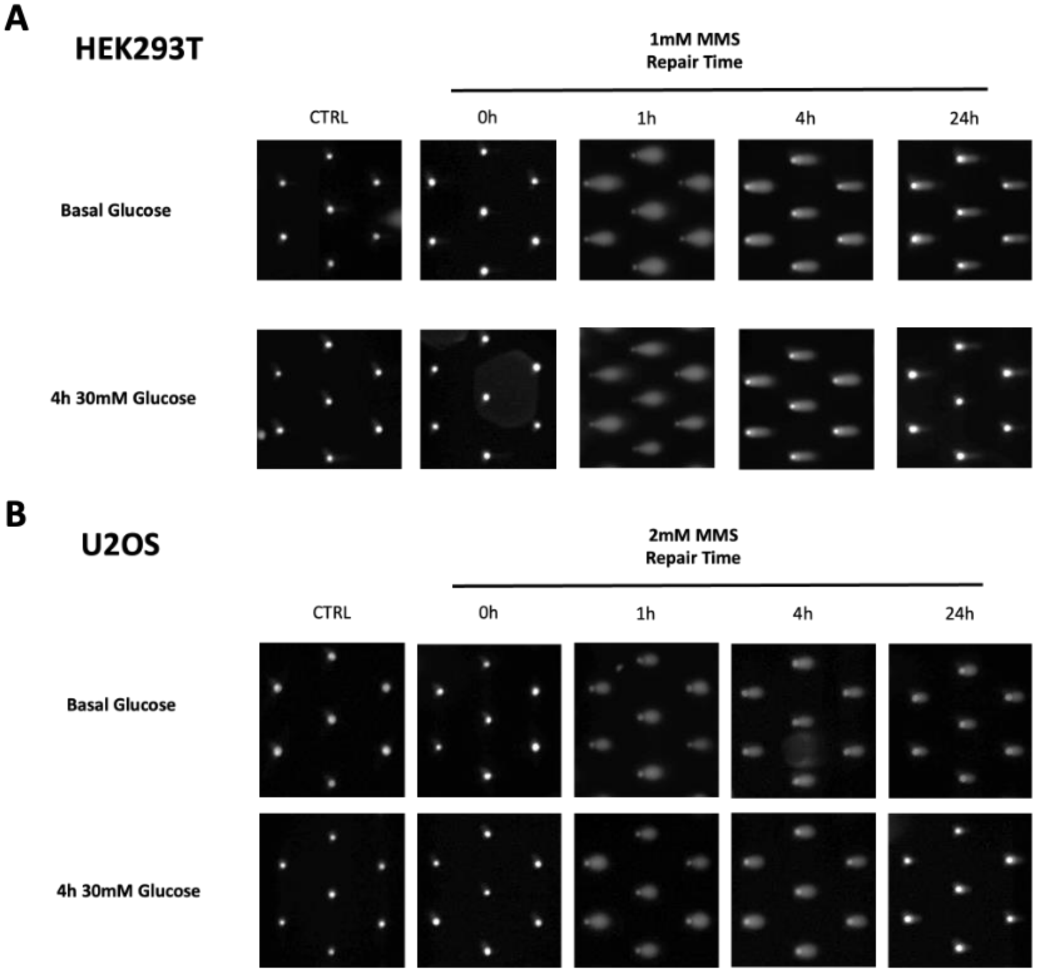

Figure S3. Comet Chip analysis following MMS exposure. (**A**) Representative Comet Chip images for basal glucose HEK293T and 4 h pre-treatment with high glucose (30 mM) HEK293T followed by 1mM MMS exposure with 0, 1, 4, 24 h of repair time. (**B**) Representative Comet Chip images for basal glucose U2OS and 4 h pre-treatment with high glucose (30 mM) U2OS followed by 2 mM MMS exposure with 0, 1, 4, 24 h of repair time.

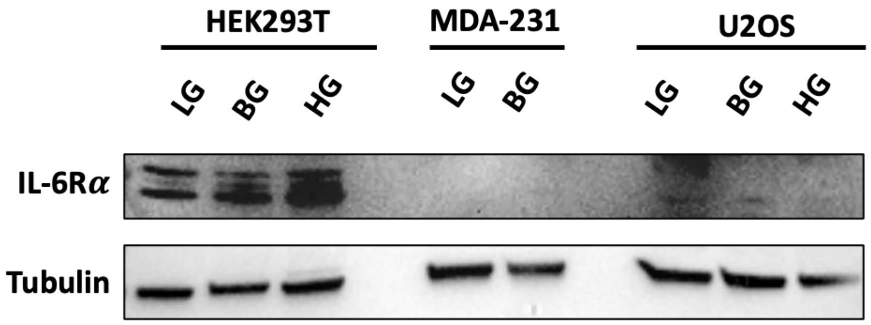

Figure S4: Immunoblot with increased exposure time showing IL-6Rα expression in U2OS and MDA-231 following harsh strip and re-probe.

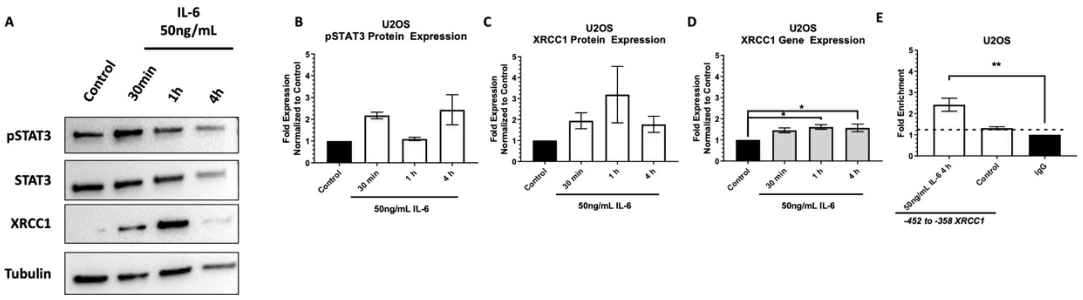

Figure S5. Acute IL-6 exposure in basal glucose U2OS. (**A**) Representative immunoblot for pSTAT3, STAT3, and XRCC1 following 50 ng/mL IL-6 exposure for 30 min, 1, and 4 h, tubulin as the loading control. (**B**) pSTAT3 protein following 50 ng/mL of IL-6 exposure for 30 min, 1, and 4 h. pSTAT3 levels are normalized to the loading control, then to the control, untreated. (**C**) XRCC1 protein expression following 50 ng/mL of IL-6 exposure for 30 min, 1, and 4 h. (**D**) *XRCC1* gene expression following 50 ng/mL of IL-6 exposure for 30 min, 1, and 4 h. (**E**) ChIP analysis of STAT3 occupancy of the *XRCC1* promoter following 50 ng/mL of IL-6 exposure for 4 h. * $p < 0.5$, ** $p < 0.01$

Table S1. IL-6 stimulation of XRCC1 protein, gene expression and STAT3 occupancy in HEK293T, MDA-231 and U2OS

XRCC1 Protein Expression	Control	30 min	1 h	4 h
HEK293T	1 ± 0	1.6 ± 0.33	1.7 ± 0.17	3.0 ± 0.20
MDA-231	1 ± 0	1.5 ± 0.16	1.15 ± 0.15	1.2 ± 0.056
U2OS	1 ± 0	1.6 ± 0.36	3.5 ± 0.42	3.3 ± 0.59
**XRCC1 Gene Expression**	**Control**	**30 min**	**1 h**	**4 h**
HEK293T	1 ± 0	1.5 ± 0.13	1.6 ± 0.13	1.8 ± 0.15
MDA-231	1 ± 0	1.8 ± 0.25	1.7 ± 0.14	1.6 ± 0.14
U2OS	1 ± 0	1.5 ± 0.14	1.7 ± 0.091	1.6 ± 0.20
**STAT3 Occupancy of the *XRCC1* promoter**	**Control**	**30 min**	**1 h**	**4 h**
HEK293T	1.49 ± 0.28	----	----	2.20 ± 0.26
MDA-231	2.4 ± 0.11	----	----	----
U2OS	1.3 ± 0.034	----	----	2.4 ± 0.27
